# The Branching and Innervation Pattern of the Radial Nerve in the Forearm: Clarifying the Literature and Understanding Variations and Their Clinical Implications

**DOI:** 10.3390/diagnostics10060366

**Published:** 2020-06-02

**Authors:** F. Kip Sawyer, Joshua J. Stefanik, Rebecca S. Lufler

**Affiliations:** 1 Department of Medical Education, Tufts University School of Medicine, Boston, MA 02111, USA; rebecca.lufler@tufts.edu; 2 Department of Anesthesiology, Stanford University School of Medicine, Stanford, CA 94305, USA; 3 Department of Physical Therapy, Movement and Rehabilitation Science, Bouve College of Health Sciences, Northeastern University, Boston, MA 02115, USA; j.stefanik@neu.edu

**Keywords:** radial nerve, variation, order of innervation, posterior interosseous nerve, superficial branch of radial nerve, forearm

## Abstract

Background: This study attempted to clarify the innervation pattern of the muscles of the distal arm and posterior forearm through cadaveric dissection. Methods: Thirty-five cadavers were dissected to expose the radial nerve in the forearm. Each muscular branch of the nerve was identified and their length and distance along the nerve were recorded. These values were used to determine the typical branching and motor entry orders. Results: The typical branching order was brachialis, brachioradialis, extensor carpi radialis longus, extensor carpi radialis brevis, supinator, extensor digitorum, extensor carpi ulnaris, abductor pollicis longus, extensor digiti minimi, extensor pollicis brevis, extensor pollicis longus and extensor indicis. Notably, the radial nerve often innervated brachialis (60%), and its superficial branch often innervated extensor carpi radialis brevis (25.7%). Conclusions: The radial nerve exhibits significant variability in the posterior forearm. However, there is enough consistency to identify an archetypal pattern and order of innervation. These findings may also need to be considered when planning surgical approaches to the distal arm, elbow and proximal forearm to prevent an undue loss of motor function. The review of the literature yielded multiple studies employing inconsistent metrics and terminology to define order or innervation.

## 1. Introduction

Knowledge of the order of motor nerve branching from main nerve trunks into skeletal muscles is of clinical importance when evaluating motor and sensory deficits, treating nerve entrapment and predicting the timing of recovery after nerve injury [1]. Radial nerve entrapments, such as radial tunnel and posterior interosseous nerve syndromes, are common and result in reduced quality of life and ability to carry out activities of daily living [2].

Numerous sites of radial nerve entrapment have been identified in the literature—five for the posterior interosseous nerve alone—each resulting in a unique clinical presentation specific to the nerve’s motor branching pattern [3]. It is imperative that clinicians have thorough knowledge of the radial nerve’s course and branching in order to provide accurate and timely diagnosis.

Although some parametric studies of the radial nerve focused on the “order of innervation” in the forearm have been carried out, they have typically defined this term poorly and employed dissimilar metrics to represent it, making it difficult to compare studies [4,5]. Specifically, some authors have identified the order of motor branching from the radial nerve when determining the “order of innervation,” while others have identified the order with which each muscle exhibits a motor entry point (the point at which a motor nerve first enters the muscle). Only a single study, carried out by Linell in 1921, documented both the branching order and motor entry order for the entire course of the radial nerve in the forearm, with findings indicating that those two orders are not always in agreement [6]. Thus, the methodological discrepancies present in modern studies prevent accurate comparisons and render them individually unable to present a complete picture of the radial nerve’s motor branching pattern.

Beyond the knowledge of an archetypal “pattern” of the radial nerve and its branches in the forearm, an understanding of common variations is of particular clinical utility vis à vis preventing the iatrogenic loss of function or initial misdiagnosis of a lesion in a patient whose presenting symptomatology or underlying anatomy may be aberrant. Some existing studies have identified cases of innervation of the brachialis muscle by the radial nerve [4,7,8], as well as motor innervation by the superficial branch of radial nerve, which is often considered purely sensory [4,5,8]. These findings have thus far not been incorporated into most anatomical texts and remain relatively unknown.

This mixed methods study aimed to provide qualitative and quantitative data of both educational and clinical significance on the branching pattern and motor distribution of the radial nerve. The primary objectives of this study were to: (1) determine the branching order of the radial nerve in the distal arm and forearm; (2) determine the order of innervation of the radial nerve in the distal arm and forearm based on the distance to motor entry points; and (3) to identify the muscular territories of each branch of the radial nerve and quantify any variation thereof. The comprehensive measurement of both branching points and motor entry points set forth here provides a more complete and specific set of data than is currently available, as these parameters had previously been used independently to describe the nerve’s anatomy or “order of innervation” [4,5]. These data will not only clarify existing reports in the literature, but also set forth an understanding of where and how branches divide from the radial nerve and reach their target structures.

## 2. Materials and Methods

### 2.1. Cadaveric Study Sample

This study utilized a sample of 35 embalmed cadavers housed in the gross anatomy laboratory at Tufts University School of Medicine that were concurrently being dissected as part of the medical and dental gross anatomy courses. One upper extremity from each cadaver was included. The selection of the laterality of each dissection was based on the fact that students performed a deep dissection of only one upper extremity, leaving the other one grossly intact and thus available for research purposes. As such, there was an inclusion of both right and left upper extremities. Cadaveric research of this nature does not require the Institutional Review Board or ethics approval at Tufts University.

### 2.2. Measurement of Radial Nerve

The 35 upper extremities were dissected initially to reveal the superficial musculature and then further to distally expose the radial nerve from the level of the spiral groove of the humerus proximally to the arcade of Frohse (supinator arch). With the extremity in anatomical position, the medial and lateral humeral epicondyles were palpated and a line was drawn bisecting them (the transepicondylar line, TEL). A pin was placed through the radial nerve at the point where it crossed the TEL to both fix it in place and serve as a consistent marker for measurement. Each muscular branch of the nerve was identified and the distance from the TEL to the nerve’s branch point from the radial nerve (“branch distance”) was measured to the nearest one-hundredth of a millimeter (0.01 mm) using a digital caliper (Neiko Tools^®^, Taipei, Taiwan). The branch points proximal to the TEL were recorded as negative values and those distal were recorded as positive. The length of each branch (a straight line from its exit from the radial nerve to its motor entry point—MEP—at the muscle) was then measured in the same fashion. The branch distance and branch length were then summed to determine the “MEP distance.” This was first performed for brachialis (B; if innervated supinator (S). The supinator was then reflected to allow for the dissection of the nerves supplying extensor carpi ulnaris (ECU), extensor digitorum and digiti minimi (ED, EDM), abductor pollicis longus (APL), extensor pollicis longus and brevis (EPL, EPB) and extensor indicis (EI).

Previous studies have used differing and often poorly defined terminology when discussing the distal branches of the radial nerve. In this study the deep branch of the radial nerve (DBRN) was defined as the nerve that remains after the superficial branch of the radial nerve (SBRN) branches from the radial nerve, while the posterior interosseous nerve (PIN) was defined as the continuation of DBRN distal to the distal border of the supinator. This is consistent with the definition used by Moore et al. in *Clinically Oriented Anatomy* [9].

### 2.3. Analysis of Radial Nerve Branches

The most common source of innervation for each muscle was determined based on the frequency with which each named branch of the radial nerve supplied it. A “most common branching order” was determined based on the mean branch distance for each muscle across all specimens. A “most common MEP order” was then determined based on the mean distance to the first MEP for each muscle. For both of these variables a one-way analysis of variance (ANOVA) followed by a post-hoc Tukey’s test was used to evaluate the differences between the muscles. Significance was set to *p* < 0.05. Studies since Linell’s in 1921 have used either one or the other of these two variables when defining the “order of innervation”; this study notably investigates both as we believe they each contribute to the conceptualization of an overall order of innervation and serve unique purposes in different clinical contexts.

## 3. Results

### 3.1. Branching Order of the Radial Nerve in the Forearm

Thirty-five cadavers, 14 of which were female and 21 of which were male, with a mean age of 79.9 ± 11.5, were included in this study. The muscular nerve branching order was determined based on the mean distance from the TEL to the first branch exiting off the main trunk to serve each muscle (branch distance, Table 1). The branching order is presented as follows, with (*) representing a significant difference in the mean branch distance (*p* ≤ 0.05): brachialis, * brachioradialis, * ECRL,* ECRB, supinator,* ED, ECU, APL, EDM,* EPB,* EPL, EI. The mean branch distance was not statistically significantly different between the ECRB and the supinator; the ED and ECU; any of the ECU, APL and EDM; or the EI and EPL. The ECRB’s branch arose proximal to that of supinator in 27 specimens (77.1%); ED’s was proximal to ECU’s in 24 (68.6%); ECU’s was proximal to APL’s in 33 (94.3%); ECU’s was proximal to EDM’s in 28 (80.0%); and EDM’s was proximal to APL’s in 18 (51.4%). The branch to the EPL arose proximal to that to the EI in 10 (28.6%) and the two arose co-terminally in 22 (62.9%).

### 3.2. Motor Entry Point Order

The distance from the TEL to the most proximal entry point of a nerve into each muscle (MEP distance) is depicted in Table 2. That order is as follows, with (*) representing a significant difference in the mean MEP distance (*p* ≤ 0.05): brachialis, * brachioradialis, * ECRL, * supinator, ECRB,* ED, ECU, EDM, APL,* EPL, EPB,* EI. The mean distance to the MEP was not statistically significant between the supinator and ECRB; ED and ECU; EDM and APL; or the EPL and EPB. The supinator’s MEP was proximal to the ECRB’s in 27 (77.1%); ED’s was proximal to ECU’s in 25 (71.4%); EDM’s was proximal to APL’s in 24 (68.6%); EPL’s was proximal to EPB’s in 20 (57.1%); EPB’s was proximal to EI’s in 23 (65.7%).

### 3.3. Motor Innervation Territories

**Figure 1:**
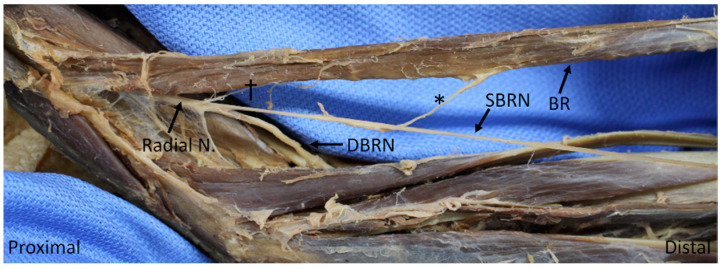
Nerve to the brachioradialis (BR) arising from the superficial branch of a radial nerve (*), with an additional smaller branch arising from the radial nerve proper (†). BR: brachioradialis; SBRN: superficial branch of the radial nerve; DBRN: deep branch of the radial nerve.

**Figure 2:**
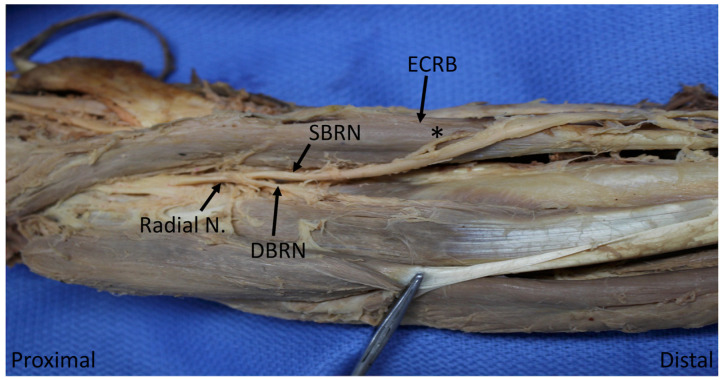
Nerve to extensor carpi radialis brevis (*) arising from the superficial branch of the radial nerve. ECRB: extensor carpi radialis brevis; SBRN: superficial branch of radial nerve; DBRN: deep branch of radial nerve.

Descriptive statistics of muscular territories are shown in Table 3. Brachialis received a branch from the radial nerve proper in 21 specimens (60%). Brachioradialis and ECRL were always innervated by the radial nerve proper, although brachioradialis also received innervation by a much more substantial branch from the SBRN in one specimen (2.9%) (Figure 1); the ECRL was solely supplied by radial nerve in all specimens (100%). The ECRB received sole innervation from the radial nerve in nine specimens (25.7%), DBRN in 16 (45.7%) and the SBRN in eight (22.9%, Figure 2); it received dual innervation from the radial nerve and DBRN in one specimen (2.9%). Supinator was generally innervated by DBRN (89%), but occasionally was served more proximally by the radial nerve (11%). The ED was innervated by PIN in 30 specimens (85.7%), DBRN in four (11.4%) and by the two nerves together in one (2.9%) The following muscles were generally innervated by PIN: ECU (82.9%), EDM (97.1%), APL (91.4), EPB (97.1%); they were otherwise supplied by DBRN (17.1%, 2.9%, 8.6%, 2.9%, respectively). EPL and EI were both exclusively innervated by PIN in all 35 specimens (100%).

### 3.4. Landmarks

With the TEL located at 0.00 mm in all the specimens, the division of the radial nerve into the superficial branch of the radial nerve (SBRN) and deep branch of the radial nerve (DBRN) occurred at a mean of 9.79 ± 12.2 mm distally. The arcade of Frohse (supinator arch) occurred at 42.3 ± 7.94 mm. The distal margin of the supinator, where the DBRN becomes the PIN, occurred at 78.6 ± 8.67 mm from the TEL.

## 4. Discussion

### 4.1. Branching and Motor Entry Point Orders

The order with which branches diverged from the main trunk of the radial nerve to serve the forearm extensors exhibited an overarching and relatively predictable pattern in most specimens. Deviation from the norm was notable in specific, consistent pairs (and one triplet) of muscles—often of the deep layer—whose branches typically exited near one another and thus were prone to swapping. This is evidenced by the statistically insignificant difference in the mean branch point distances between the ECRB and supinator; ED and ECU; ECU and APL and EDM; and the EI and EPL. There was similar variation in the MEP order, with statistically insignificant differences between the ECRB and supinator; ED and ECU; EDM and APL; and the EPL and EPB.

The overall MEP order found here is consistent with that described by Abrams et al., who used this metric to define the “order of innervation” [4]. Branovacki et al. produced a descriptive (i.e., not quantitative) account of the most common branch order in sixty specimens that agrees with the overall findings on the branching order here [5]. Lastly, although Mazurek and Shin did not explicitly report how they defined their “most commonly accepted order of innervation,” their list does agree with this investigation’s findings on the branching order [2].

Knowledge of the order in which muscles are innervated by the radial nerve, along with the length of individual branches, has important clinical implications. The shortest distance along a nerve trunk and the subsequent muscular branch (represented in this study by the MEP distance) is an important predictor of the order and timing of re-innervation (and thus the recovery of individual muscle function) after nerve injury [1,4]. Furthermore, the typical order in which nerves branch to serve muscles (here represented by branch distance) is an important factor to consider when localizing lesions in cases of nerve entrapment [2]. This information is particularly helpful when used in conjunction with knowledge of the locations of structures which are known to cause entrapment, such as the arcade of Frohse, as the relative location of these structures to individual motor branches can inform specific localization. Lastly, when understood together, branch order and MEP distance can aid in conceptualizing the overall course of the motor branches, which is important when dissecting in the vicinity of the nerve during surgery or planning for nerve transfers [8,10].

### 4.2. Innervation of Brachialis by Radial Nerve

This study noted the partial innervation of brachialis by the radial nerve in 60% of specimens, with the branch arising from the radial nerve at a mean of 66.4 ± 19.6 mm proximal to the TEL, in the plane between the brachialis and brachioradialis muscles. This is consistent with the findings of previous studies in Western populations that have documented rates of 50% [4], 65% [8] and 67% [7], and lower than the rates of 81.6% [11] and 100% [12] documented in Asian populations; Blackburn et al. hypothesized that part of this discrepancy may be due interracial differences [7]. As this branch from the radial nerve—present in potentially two-thirds of patients—travels in the plane between the brachialis and brachioradialis, care should be taken when surgically dissecting these two muscles apart (such as in an anterior approach to the humerus) to prevent the denervation of the lateral portion of the brachialis [7,11].

A survey of the six most commonly used preclinical anatomical texts at Tufts University School of Medicine—*Atlas of Human Anatomy*, 6th edition [13]; *Clinically Oriented Anatomy*, 7th edition [9]; *Essential Clinical Anatomy*, 5th edition [14]; *Grant’s Atlas of Anatomy*, 13th edition [15]; *Gray’s Anatomy for Students*, 3rd edition [16]; *Atlas of Anatomy*, 2nd edition [17]—yielded no mention of this common source of innervation. Given that the results of this study support the results of previous studies that the brachialis muscle is partially innervated by the radial nerve at a greater than 50% incidence rate, it can be argued that this should be regularly included in all textbooks and atlases, as well as in medical student anatomy curricula.

### 4.3. Muscular Innervation by Superficial Branch of Radial Nerve

The superficial branch of the radial nerve was found to provide sole innervation to ECRB in 25.7% of specimens in this study (Figure 2). The breakdown of the innervation source for the ECRB was remarkably consistent with that found by Abrams et al., who documented innervation rates of 25% by SBRN, 30% by radial nerve and 45% by what the authors termed PIN (which we would refer to as the DBRN as it has not yet reached the distal border of supinator; see below) [4]. These findings are noteworthy given that there exists a commonly held notion that the SBRN is a purely sensory nerve and serves no motor function. As above, none of the six textbooks surveyed discussed this common variant, and many reinforced the teaching that SBRN is sensory-only.

Furthermore, the current study sample also included one specimen with what appeared to be the primary innervation of the brachioradialis by SBRN (with an additional, much less substantial branch from the radial nerve, Figure 1 above). A search of the existing literature revealed no other studies that have reported such an anomaly. A limitation to this study is that it cannot confirm that this nerve from SBRN carried motor fibers, although its bulky appearance and the lack of other significant nerve supply was quite suggestive of this.

Knowledge of the potential for innervation of either the ECRB or brachioradialis muscles by the SBRN would be of significant importance in many surgical settings. Given the SBRN’s very superficial location under the skin and lack of robust protection, it is susceptible to iatrogenic injury during various surgical approaches, including the commonly performed radial forearm free flap for the reconstruction of tissue defects, where overlying tissue is harvested while the SBRN is ideally left intact [18,19]. It is well known that injury to the distal portion of the nerve can result in intractable pain or neuroma formation [20], but there has been less focus on the potential for proximal injury, let alone the possible consequence of muscular denervation that such injury could entail. Although the SBRN is more proximally protected thanks to the overlying mobile wad, it is still susceptible to injury or entrapment due to surgical dissection, post-surgical edema or scarring, or—in a case of parathyroid autotransplantation followed post-operatively by one of the authors (F.K.S.)—delayed transplanted gland hypertrophy. The knowledge that SBRN has a motor component in nearly a third of the studied specimens should prompt heightened concern for the preservation of the nerve if at all possible.

### 4.4. Inconsistency of Nomenclature and Territories

Through the course of reviewing existing literature, it became apparent that a disparity exists in the naming conventions for the DBRN and its successor, PIN, between clinicians and anatomists. Most notably, literature authored by physicians typically did not distinguish DBRN from PIN at all. Instead, the nerve that remains after the exit of SBRN from the radial nerve was referred to exclusively as the PIN by multiple authors [2,4,5]. This is in contrast to anatomical textbooks; for instance, *Clinically Oriented Anatomy* references the division of the radial nerve into the SBRN and DBRN, and describes the course of the latter as follows:

“the deep branch of the radial nerve, after it pierces the supinator, runs in the fascial plane between superficial and deep extensor muscles in close proximity to the posterior interosseous artery; it is usually referred to as the posterior interosseous nerve” [9].

*Terminologia Anatomica* agrees, recognizing a “deep branch” of the radial nerve that terminates in the “posterior interosseous nerve” [21]. It is unclear as to why clinicians deviate from this paradigm and combine DBRN into PIN; regardless, it leads to lack of clarity and represents a discrepancy between basic science and clinical terminology that would ideally be reconciled.

## 5. Conclusions

Although the radial nerve does exhibit a noteworthy degree of variability in the posterior forearm, in terms of both branch territories and order of innervation, this investigation identified a pattern that was overall quite predictable. The results here serve to provide a more cohesive view of the morphologic parameters and patterning of the radial nerve and its motor branches in the distal arm and forearm than has been offered in the existing literature and to bring to further light some notable variations have often been overlooked. Given that injury to, or disease of, the radial nerve can present significant functional limitations that negatively affect quality of life [22], it is imperative that the relevant clinicians understand the nerve’s anatomy and its potential for variation in thorough detail. These findings will ideally help to inform clinicians in their approach to the examination and diagnosis of radial nerve pathology, as well as in the safe performance of surgical dissection in the nerve’s vicinity.

## Figures and Tables

**Table 1:** Branching order, based on the mean distance in mm along the trunk from the transepicondylar line to the first branch to a muscle. *p* values are listed for the comparison of a mean to the one below it. *p* values comparing any muscle to another muscle not adjacent to it on this table were all <0.05, except for the extensor carpi ulnaris (ECU) and extensor digiti minimi (EDM) (*p* = 0.1281).

Muscle	N	Mean	Standard Deviation	*p* (To Next Muscle)
Brachialis	21	−66.41	19.63	<0.0001 *
Brachioradialis	35	−40.37	12.08	0.0036 *
Extensor carpi radialis longus	35	−26.02	10.53	<0.0001 *
Extensor carpi radialis brevis	35	17.88	18.29	0.9553
Supinator	35	22.78	11.56	<0.0001 *
Extensor digitorum	35	81.90	12.03	0.9996
Extensor carpi ulnaris	35	84.60	10.60	0.1337
Abductor pollicis longus	35	94.92	15.26	1.0
Extensor digiti minimi	35	94.99	15.99	<0.0001 *
Extensor pollicis brevis	35	112.44	21.28	0.0271 *
Extensor pollicis longus	35	124.75	16.48	1.0
Extensor indicis	35	126.84	16.66	Not Applicable

* Indicates significant difference in mean branching distance between the listed muscle and the muscle below it (i.e., more distally) on the chart.

**Table 2:** Motor entry point order, based on the mean distance in mm from the transepicondylar line to the entry into the muscle (sum of branch distance, above and branch length). *p* values are listed for the comparison of a mean to the one below it. *p* values comparing any muscle to another muscle not adjacent to it on this table were all <0.05.

Muscle	N	Mean	Standard Deviation	*p* (To Next Muscle)
Brachialis	21	−52.22	23.19	<0.0001 *
Brachioradialis	35	−20.22	17.11	<0.0001 *
Extensor carpi radialis longus	35	5.10	10.04	<0.0001 *
Supinator	35	47.67	8.42	0.4593
Extensor carpi radialis brevis	35	57.13	19.60	<0.0001 *
Extensor digitorum	35	94.24	13.68	0.9896
Extensor carpi ulnaris	35	99.08	13.28	0.0017 *
Extensor digiti minimi	35	116.33	17.39	0.8526
Abductor pollicis longus	35	123.38	13.15	<0.0001 *
Extensor pollicis longus	35	147.56	21.91	0.5003
Extensor pollicis brevis	35	154.22	22.24	0.0062 *
Extensor indicis	35	163.47	20.40	Not Applicable

* Indicates significant difference in mean motor entry point distance between the listed muscle and the muscle below it (i.e., more distally) on the chart.

**Table 3:** Percentage distribution of each muscle among the branches of the radial nerve (e.g., muscular territories). Note: percentages for the brachioradialis (BR), the extensor carpi radialis brevis (ECRB) and the extensor digitorum (ED) sum to greater than 100% as certain specimens had dual innervation to these muscles.

Muscle	Radial Nerve	Superficial Branch of the Radial Nerve	Deep Branch of the Radial Nerve	Posterior Interosseous Nerve
Brachialis	60			
Brachioradialis	100	2.86		
Extensor carpi radialis longus	100			
Extensor carpi radialis brevis	28.57	25.71	48.57	
Supinator	11.43		88.57	
Extensor digitorum			14.29	88.57
Extensor carpi ulnaris			17.14	82.86
Extensor digiti minimi			2.86	97.14
Abductor pollicis longus			8.57	91.43
Extensor pollicis brevis			2.86	97.14
Extensor pollicis longus				100
Extensor indicis				100
